# Sustainable emerging high-intensity sonication processing to enhance the protein bioactivity and bioavailability: An updated review

**DOI:** 10.1016/j.ultsonch.2023.106464

**Published:** 2023-06-01

**Authors:** Muhammad Hussain, Munkh-Amgalan Gantumur, Muhammad Faisal Manzoor, Kifayat Hussain, Jie Xu, Rana Muhammad Aadil, Abdul Qayum, Ishtiaq Ahmad, Hao Zhong, Rongfa Guan

**Affiliations:** a College of Food Science and Technology, Zhejiang University of Technology, Hangzhou 310014, China; b Key Laboratory of Marine Fishery Resources Exploitment & Utilization of Zhejiang Province, China; c Food College, Northeast Agricultural University, No. 600 Changjiang St. Xian fang Dist, 150030 Harbin, China; d Guangdong Provincial Key Laboratory of Intelligent Food Manufacturing, Foshan University, Foshan 528225, China; e School of Food Science and Engineering, South China University of Technology, Guangzhou 510641, China; f Departments of Animal Nutrition, Institute of Animal and Dairy Sciences, University of Agriculture Faisalabad, Pakistan; g National Institute of Food Science and Technology, University of Agriculture, Faisalabad 38000, Pakistan; h School of Food and Biological Engineering, Jiangsu University, 301 Xuefu Road, Zhenjiang, Jiangsu 212013, China

**Keywords:** Allergenicity, Bioactive compounds, Anti-nutritional factors, Functional attributes

## Abstract

High-intensity ultrasound (HIU) is considered one of the promising non-chemical eco-friendly techniques used in food processing. Recently (HIU) is known to enhance food quality, extraction of bioactive compounds and formulation of emulsions. Various foods are treated with ultrasound, including fats, bioactive compounds, and proteins. Regarding proteins, HIU induces acoustic cavitation and bubble formation, causing the unfolding and exposure of hydrophobic regions, resulting in functional, bioactive, and structural enhancement. This review briefly portrays the impact of HIU on the bioavailability and bioactive properties of proteins; the effect of HIU on protein allergenicity and anti-nutritional factors has also been discussed. HIU can enhance bioavailability and bioactive attributes in plants and animal-based proteins, such as antioxidant activity, antimicrobial activity, and peptide release. Moreover, numerous studies revealed that HIU treatment could enhance functional properties, increase the release of short-chain peptides, and decrease allergenicity. HIU could replace the chemical and heat treatments used to enhance protein bioactivity and digestibility; however, its applications are still on research and small scale, and its usage in industries is yet to be implemented.

## Highlights

• High-intensity ultrasound is recently used in protein modification.
• Various edible proteins are successfully ultrasonicated to enhance their bioactivity and bioavailability.
• Ultrasonication can also substantially decrease the allergenicity and anti-nutritional factors in allergy-causing proteins.
• Besides the various advantages of ultrasound in protein modification, it is still not universal and has limitations.

## 1 Introduction

The term (protein bioactivity and functionality) is defined as the property of protein that benefits the body system and overall human health and how a protein behaves as a colloidal biopolymer in a food system with or without other food ingredients. It also includes extrinsic, intrinsic, physicochemical and nutritional attributes [1], [2]. The bioactive properties of proteins are related to human health; proteins with high bioactivity are considered functional proteins that can release bioactive peptides and possess numerous health benefits, including oxidative, anti-cancer, antihypertensive, anti-inflammatory, and antimicrobial [3].

Any protein's bioavailability, functionality, and bioactivity define its quality; proteins with better functional and bioactive attributes are considered high quality [4]. Bioactive and bioavailable properties of proteins are related to product formation; some common functional properties of a protein are solubility, foaming ability, emulsifying activity, gelation, film formation, and viscosity. Bioactive properties in proteins are controlled by numerous factors, including secondary and tertiary structure, surface hydrophobicity, isoelectric point, and molecular weights [5], [6].

Both the bioactivity and digestibility of a protein are essential for use in different foods and can be modified by different techniques according to the desire. [7] Proteins are complex molecules, and their functionality modification is very interesting and vital in the food industry. Knowing the structural properties and techniques to modify a specific functionality [7], [8]. Different techniques, including pH adjustment, enzymatic hydrolysis, high temperature, and conjugation, are used to modify the bioactivity, bioavailability, digestibility, and functionality of protein, but most of these techniques also have adverse outcomes [9]. Ultrasonication is a recent novel green technique recently applied successfully to enhance the bioactivity and functionality of plant- and animal-based proteins [10].

Ultrasound comprises auto-static high-frequency (20 kHz) sound waves produced from the molecular vibration that vacillates in a propagation pendulum [11], [12]. Recently ultrasound has been efficiently applied in different food industries substituting traditional, and chemical approaches [13], [14], [15]. Ultrasound offers new ways of producing bioactive peptides and improving the bioactive properties of proteins. During ultrasonication, the transmission of sound waves oscillates through the Food, leading to pressure fluctuations, resulting in the formation and collapse of bubbles, a phenomenon called cavitation. This cavitation produces localized shear force, temperature, and pressure changes, which alters Food's physical and chemical properties [16].

The versatility of ultrasonication has opened new windows to an extensive range of food applications, including emulsification, production of nanoparticles, and bio-component separation inactivation of microorganisms and enzymes [17]. For protein quality enhancement, ultrasonication is gaining interest as an alternative to chemical and thermal treatment. The bioactivity and functional attributes of plant- and animal-based proteins have been successfully enhanced recently; it also helped release bioactive peptides [2], [18].

Recently, there has been an increasing demand for high-quality protein, which drives the exploration of alternative methods to chemical methods. The eco-friendly, less economical, and easy-handling properties of ultra-sonication make it an exclusive technique to enhance the bioactivity and functionality of proteins.

Recently some review papers have been published entitled “Direct Contact Ultrasound in Food.

Processing: Impact on Food Quality“ [19], ”Application of High-Intensity Ultrasound to Improve Food Processing Efficiency: A Review“ [20]. However, to the best of our knowledge, there is no data available regarding the application of ultrasonication to improve the bioactivity and digestibility of protein. Therefore the present review emphasizes on impact of ultrasound on bioactive properties, peptides release, digestibility, allergenicity, and functional properties. Additionally, it will also discuss its impacts on anti-nutritional factors.

## 2 Application of H.I.U. in food processing

The application of non-chemical green technologies in food processing is getting more critical, especially in processing bioactive compounds and functional foods [21]. Due to its eco-friendly and wide application, ultrasonication has revolutionized the food industry. This green technology offers numerous advantages, including retention of bioactive compounds, enhanced process efficiency, high-quality product, and shelf life [22], [23], [24]. In food processing, HIU has many advantages. It can extract phytochemicals and peptides without modifying their organoleptic properties and bioactivities. HIU also reduces the microbial load in the finished product due to its bactericidal effect [25], [26]. The application of HIU in Food exploits both chemical and mechanical effects; these effects result from different frequencies used. The mechanical effect occurs at low frequencies (20–100 kHz) due to the bubbles formation and cavitation [27]. HIU treatment on medium frequencies predominantly has chemical effects due to acoustic flow and rapid formation and collapsing of bubbles [28]. In food processing, HIU can be employed for various operations, including the formation of emulsions, cutting, fruit drying, meat tenderization, thawing, filtration, extraction and many other techniques. The application of ultrasonication in food processing is summarized in Table 1.

**Table 1:** Summarizing the application of HIU in food processing and preservation.

Technique	Target food	Frequency and time	Mechanism/impact	Ref
Emulsion formation	Myofibril emulsion	650 W, 20 kHz, 6 min	Exposure of sulfhydryl groups, formation of a stable emulsion	[145]
Extraction of bioactive compounds	Spondias purpurea L.peel	200w, 20 kHz, 15 min	Softening of tissues, High antioxidant ciriguela peel extract	[146]
Meat tenderization	Meat	110 W, 40 kHz, 60 min	releasing myofibrillar proteins	[147]
allergenicity reduction	Dairy proteins	300 W, 20 kHz, 15 min	IgG/IgE binding ability, 60 % reduction in allergenicity	[116]
Crystallization	Fotagliptin benzoate	80 W, 20 kHz, 4 min	Reduced crystallization time and increased crystal properties	[148]
Bacterial inactivation	Fresh tomato juice	400 W, 20 kHz	Rupturing of microbial structure, Free from spoilage microorganisms up to 10 days	[149]
Osmotic drying	Persimmon fruit	35 kHz for 30 min	Increase in pore size, Drying time reduced by 33%	[150]
Extraction of aroma				[151]
Modification of enzymatic activity	Lipase enzyme	150 W for 15 min	changes the characteristics of enzymes	[152]
Peptides extraction	Antioxidant peptides	400 W	increases the enzyme's accessibility to the peptide	[153]

Regarding protein processing via ultrasonication, both sono-mechanical and sonochemical impact the protein structure. HIU mechanical effect modifies protein structure through molecular agitation, permanently altering the 3D structure. Similarly, the chemical effect breaks the chain and alteration amino acid side groups and exposes hydrophobic residues, thus enhancing the protein attributes [29].

In food industries, protein can be modified using HIU by changing physiological and structural properties; for instance, Hussain et al., 2022 stated that ultrasound could significantly improve both digestibility and bioactivity in potato proteins. Similarly, another research conducted by [30] showed that ultrasonication could improve the functional properties of tamarind seed protein isolates by conformational changes in the primary structure. The function of proteins is mainly associated with the 3D folded structure, and biologically active proteins must keep their structure and tend to tolerate external changes; HIU, however, modifies the primary structure without loss of any activity [29]. HIU is associated with futuristic food processing at the industrial level; various foods can be treated for different purposes, especially proteins, to enhance bioactivity and digestibility.

## 3 HIU mechanism in protein bioactivity and bioavailability enhancement

Ultrasound can produce longitudinal penetrating waves, which causes an auto-static cavitation effect. During ultrasonic treatment, auto-static cavitation is responsible for the contraction and expansion of preexisting microbubbles [31], [32]. The alteration in proteins during HIU treatment is primarily due to transient cavitation, which creates a fast creation and collapsing of bubbles at the critical points of resonance, generating high pressure of 30 MPa and an instant high temperature of about 5000 k, which induces physical, thermal and chemical impacts on the protein structure [33], [34]. The bioavailability and digestibility of proteins depend on the tertiary structure; due to the compact tertiary structure, most plant-based proteins are low in both digestibility and bioavailability [35], [36].

Similarly, the bioactivity of proteins depends on the availability of hydrophobic interaction sites and the stretching of amide bands, amide band I and amide band II [37]. Any alterations in the amide band or change in the secondary structure of a protein can modify the bioactivity and bioavailability of proteins. Ultrasonication can uncover sites of hydrophobic interactions in the protein structure, thus enhancing proteins' bioactivity [38]. For instance, in research, the authors tested the impact of H.I.U. (50, 75, and 100 % amplitude) and 52 W on millet protein fractions' bioavailability and functional properties. The results showed a maximum increase of 58 % in the bioavailability in the treated samples.

Similarly, there was a significant increase in solubility, digestibility, and foaming capacity [39]. In another study conducted by Hussain et al., 2022, they extracted and isolated potato protein and then subjected it to high-intensity ultrasound (600 W) for 10, 15, and 20 min. The results suggested a significant improvement in the bioactivity of 56 % and 20 % digestibility during *in-vitro* digestion. These changes were due to the exposure of hydrophobic residues and the shifting of amide bands during ultrasonic treatment.

**Fig. 1:**
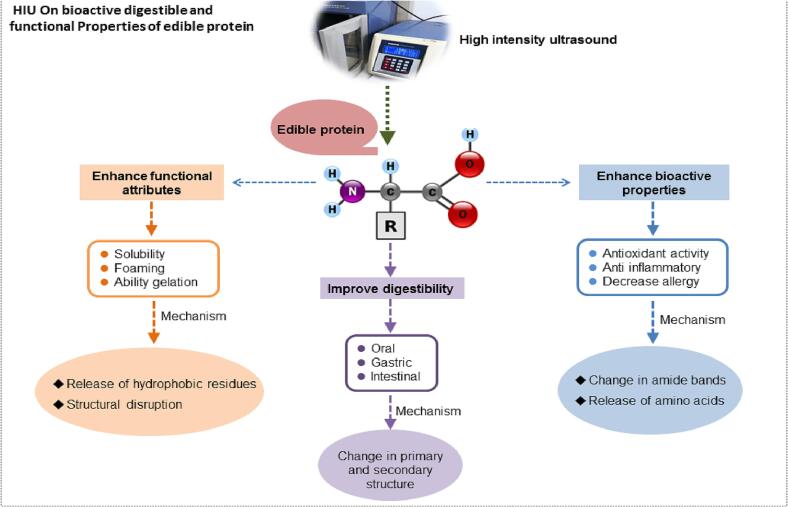
Depicting the mechanism and effect of HIU on different aspects of edible protein, including bioactivity and digestibility.

Proteins with high antioxidant activities are highly potent functional food in the nutraceutical and medicinal industries [40]. Therefore, HIU. is a green physical treatment with simple operations and mechanisms to improve proteins' bioavailability and bioactivity. The mechanism of HIU on the alteration of protein structure is summarized in Fig. 1.

## 4 Role in bioactivity

**Fig. 2:**
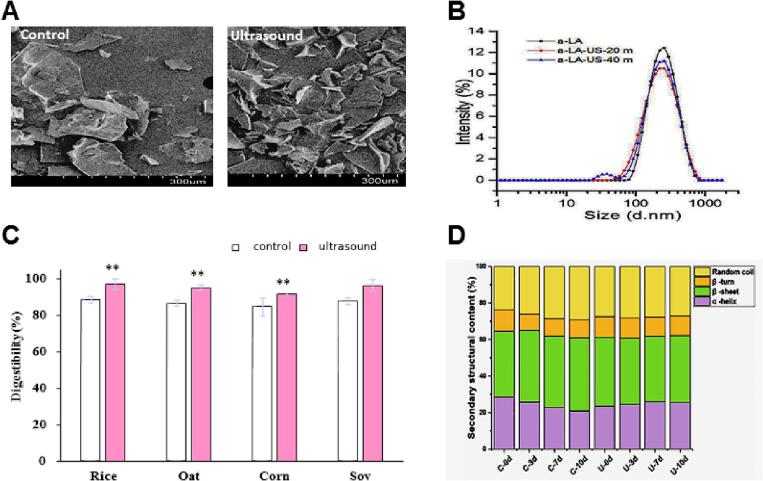
A) S.E.M. monograph comparing control and HIU (600 W 20 min) treated potato protein. B) Size reduction in LA proteins treated with HIU for 20 and 40 min. C) HIU-induced improvement in digestibility of different proteins isolated from rice, oat, corn and soy. D) Conformational structural changes induced in actomyosin complex treated with HIU. Reprinted with permission from Elsevier. Adapted from A [48], B [161], C [83], and D [162].

The bioactivity of proteins is a significant attribute in functional foods and nutraceuticals. Bioactive proteins can be vital in antioxidant, antihypertensive, antimicrobial, and other biological activities [41]. Ultrasound is an ideal technique that can substantially enhance the bioactivity of different proteins [42]. Some recent research data showing the impact of HIU on bioactivity and the structure of different proteins is summarized in Fig. 2.

### 4.1 Antioxidant activity

Hydroxyl radicals are highly reactive and readily interact with biomolecules inside a cell by diffusing through the cell membranes causing cell death. These free radicals must be destroyed to protect human health [43]. Different proteins and their peptides with high antioxidant activities are used to cure different diseases [44], [45]. Different techniques can also enhance protein antioxidant activity, including high temperatures, high pressure, chemical treatments, and ultrasonication [46], [47].

In a research conducted by [48] where they extracted purified and treated potato protein with HIU for 10, 15 and 20 min (600 W ). All treated samples showed a substantial improvement in hydroxyl radical scavenging activity (33% increases). Similarly, there was also an enhancement in D.P.P.H. radical scavenging activity (23% increase). The highest increase in both samples was at HIU treatment for 20 min; these changes were induced due to the conformational changes in protein structure [48]. Applying high-intensity ultrasound might be responsible for releasing these amino acids resulting in increased DPPH radical scavenging activity. Another reason for improving DPPH radical scavenging activity was HIU's transformation and exposure to active amino acid residues, which react with oxidants [49].

Likewise, in another research, the antioxidant activity of β-Lactoglobulin was tested, samples were treated with HIU for 10–30 min at the frequency of 20 kHz, and the antioxidant activity was investigated using spectroscopic techniques. The results revealed that HIU treatment significantly improved antioxidant activity, with a maximum enhancement in DPPH (57%) in HIU treated for (20 min). The treated samples also showed greater 2,2ʹ-Azino-bis (3-ethylbenzothiazoline-6-sulfonic acid) diammonium salt radical scavenging activity and oxygen radical absorbance capacity. The CD analysis showed an increase in β-sheet contents of β-Lactoglobulin and changes in primary and tertiary structures; SEM also showed bigger aggregates than the untreated samples [42], [50].

Natural polyphenols are a great source of dietary antioxidants that substantially improve human health; recently, coffee leaves have been used as an antioxidant agent for commercial purposes [51], [52]. In research, the impact of HIU on the antioxidant activity of peroxidase and polyphenol oxidase in coffee leaves was investigated; the results showed a significant enhancement in antioxidant activity and PPO activity; however, the incubation time gradually decreased antioxidant activities [53].

In recent research, mug bean proteins were subjected to HIU to investigate the influence on antioxidant and physicochemical properties. Mug bean isolates were treated with 546 W for 20 min. Antioxidant activity was measured through free radical scavenging activity, iron chelating ability, and reducing power, while physicochemical properties were recognized through SDS-PAGE and FTIR. Results showed a significant increase with increase in ultrasound power with values of 0.1087 (ABTS), 1.796 (hydroxyl), 1.003 (superoxide anion), and 0.185 (Fe^2+^ chelating ability) in 546 W power. The smallest particle size was measured in samples treated with 546 W, with surface hydrophobicity (367.95 AU) and zeta potential (−36.37 mV) [54]. The above mention data concludes that HIU is a reliable and favorable technique to improve antioxidant properties in proteins; in conclusion, HIU can be applied in both pilot and upgrade scales to increase the quality of proteins.

### 4.2 Antimicrobial activity

Antimicrobial proteins and peptides are generally extracted from animal and plant sources and are important in the food and pharmacology industry. These can prevent pathogenic bacteria; some peptides are also antiviral [55], [56]. In contrast to conventional antibiotics, antimicrobial peptides bind to net positively charged and destroy bacterial cell membranes, but the antibacterial activity is generally feeble due to a lack of electrostatic absorption in the bacterial cell membrane [57], [58], [59]. Numerous studies have shown the synergistic effect of antimicrobial peptides and ultrasonication against pathogenic bacteria; likewise, few studies revealed the enhancement of the antimicrobial activity of peptides treated with low-intensity ultrasound [60].

Pathogenic bacteria, especially Escherichia coli, are a leading threat to the food industry and consumers. Recently, innovative techniques, especially antimicrobial peptides, have been used to inactivate foodborne pathogens. In a research conducted by [60], [61], they treated an antimicrobial peptide TGH2 (AEFLREKLGDKCTDRHV) with ultrasound to enhance its microbial activity. The results showed that the inhibitory concentration (MIC) of TGH2 on *E. coli* decreased by 4-fold to 31.25 μg/mL under 0.3 W/cm^2^ ultrasound treatment, while the time-kill curve analysis showed that low-intensity ultrasound combined with peptide TGH2 had an enhanced synergistic bactericidal effect after 0.5 h. Structural studies by CD showed a partial untangling of α-helical structure in TGH2 during 30 min of ultrasound treatment, 0.3 W/cm^2^ [61].

Similarly, in another study, whey protein-totarol nanoparticles (natural antimicrobial compound) were treated with ultrasound, and the effect on physicochemical properties and microbial inhibiting ability on staphylococcus were studied. After ultrasonication, the particle size of the samples was reduced to 31.24 ± 5.31 to 24.20 ± 4.02 nm, with a reduction in particle distribution. The ultrasound-treated nanoparticles were subjected to cell membrane damage analysis and a time-killing assay test. Compared to untreated samples, the MIC in ultrasound-treated samples decreased from 4 to 2 μg/mL. Also, there was a significant (*p < 0.05*) decrease in killing time in treated samples. The diffusion analysis proved that inhibition zones in treated samples were 3-fold compared to untreated particles. The alteration in the bacterial cell membrane by ultrasound-treated nanoparticles showed an effective antimicrobial activity against *S. aureus*, recommended that whey protein-totarol nanoparticles treated with ultrasound were more effective than untreated nanoparticles [62].

Bacterial biofilms are major health distress causing intractable disorders like resistant infections, implant infections, and chronic wounds. Ultrasonic therapies and ultrasound-treated antimicrobial nanomaterials have recently shown promising results in obliteration biofilms [63]. To maximize the treatment and the damage of healthy tissues, a combined treatment of ultrasound and antimicrobial nanoparticles has been reported to obtain a synergistic effect. Ultrasonication enhances the microbial activity of PNPs, swiftly distorts biofilm, and enhances penetration of antimicrobial peptides resulting in a 100–1000-fold reduction in bacterial concentration [64].

### 4.3 Effect on peptides bioactivity and release

Food Bioactive peptides are obtained from plant, animal, and marine sources [65]. Bioactive peptides contain various biological functions, including antihypertensive, anti-oxidative, anti-carcinogenic, immunomodulation, and growth-regulating properties [3].

Bioactive peptides are relatively unstable, have low oral bioavailability, and have a short half-life [66], [67]. HIU is an innovative, green technology that can enhance peptide bioactivity, bioavailability, and safe delivery. Due to the generation of cavitation and bubble formation during HIU treatment, it has been reported that it can facilitate the extraction of peptides from food matrix with high yields [68], [69]. Ultrasound-treated biofilms for peptides and ultrasound-assisted encapsulation can also improve the bioavailability and stability of peptides [70].

The mechanism of ultrasonication-assisted release of antioxidant peptides was studied in a study. The gelatin solution was treated with H.I.U. (300–400 W) followed by enzymatic hydrolysis. The obtained results showed a higher degree of protein recovery rate and a higher degree of hydrolysis. Similarly, it also significantly enhanced DPPH and ABTS radical scavenging activity. The morphological analysis of treated samples showed cracks and untangling of protein; ultrasonication hinders hydrogen bonding, reduces the crosslinking between collagen molecules, and lowers the thermal stability, resulting in enhanced peptides recovery and bioactivity [71].

The number and size of small peptides in the digests represent their bioactivity, including antioxidant activity. It has been observed that with ultrasonication treatment time, an increase in peptide concentration is accompanied by an enhancement in antioxidant activity [72]. Selenium-containing peptide TSeMMM is an immunomodulatory functioning peptide mainly extracted from selenium-enriched rice protein [73]. However, its biological activity is hampered during gastrointestinal digestion. In research, TSeMMM was treated with HIU and encapsulated with zein gum to enhance its bioactivity and bioavailability. HIU treatment was done with 360 W for 5 min. During in vitro digestion, the peptide release rate was increased to 80 % in HIU-treated samples. Also, there was an improvement in the oral bioavailability of treated samples [74].

Similarly, [69] investigated the effect of HIU on antioxidant activity and ACE inhibitory activity of peptides obtained from dry-cured pork loins. Different parameters were analyzed during 42 days of storage. The results showed that compared to untreated, HIU-treated samples exhibited high antioxidant activity and ACE inhibitory activity throughout the storage time.

Green soybean pods (GSP) are agro-industrial waste products produced during milk manufacturing and soybean freezing [75]. These pods are rich in antioxidants and bioactive compounds. In recent research, ultrasound was applied to isolate flavonoids and polyphenols from soy pods. The ultrasonication-assisted yield was desirable, with the highest yield at 50% amplitude with an extraction time of 10.5 min. According to the predicted values, the extracted flavonoid content and their antioxidant activity were high with ultrasonic extraction. Procyanidins were the primary polyphenols in dried GSP crude extracts at 0.72 ± 0.01 mg/100 g [76].

## 5 Enhancement in bioavailability/digestibility

Protein bioavailability is defined as the availability of a specific protein for the human body's utilization, and protein digestibility is defined as the degradation of protein into small fragments under gastrointestinal conditions; different proteins have different digestibility [77]. A protein is considered highly bioavailable if it is easier to dissociate into amino acids and absorbs to make other proteins [78]. Protein is among the essential nutrients needed for energy and well-being, needed in large amounts daily [79]. The data regarding the impact of HIU on bioactivity and bioavailability of proteins are listed in Table 2.

**Table 2:** Presenting the effect of HIU on bioactivity, bioavailability and functional properties of different proteins.

Type of protein	HIU-Frequency	HIU-Time	Enhancement	Mechanism	Ref
Potato protein	600 W, 20 kHz,	20 min	23% OH radical scavenging activity, 12% in the digestibility	Change in Amide bands	[48]
Soy protein isolates	1000 W, 20 kHz	15 min	Increase in antioxidant activity and degree of hydrolysis	Breaking the dense structure	[38]
Milk protein isolates and whey protein isolates (WPI)	500 W 20 kHz,	10 min	Increased emulsifying activity	Modification in beta-sheet	[154]
Soybean protein isolate (SPI)	300 w, 18 kHz	18 min	Improvement in emulsion formation	Protein structural disruption	[72]
Rice brain protein and Almond protein isolate	2600 W and 5 kHz	15 min	Enhancement in foaming ability and solubility	Exposure to hydrophobic residues	[155]
Actomyosin complex	400 W, 20 kHz	10 min	increased surface hydrophobicity, and active sulfhydryl content	unfolding of molecular structure and the conformational changes	[91]
Myofibrillar protein	650 W, 20 kHz	6 min	Increased the emulsifying activity, the emulsifying stability	Unfold protein chains and decrease the particle size.	[145]
Liver protein hydrolysate	400 W, 40 kHz,	1 min	Enhancement in chelating ability and reducing power.		[156]
Chicken plasma protein	200 W, 20-kHz	30 min	Significant increase in antioxidant properties	Exposure of the hydrophobic regions	[97]
Rapeseed protein hydroxylated	600 W 20-kHz	12 min	Improved functional properties of proteins ACE inhibitory activities	By enhancing surface hydrophobicity and solubility	[18]
Millet protein concentrate	100 W, 20-kHz	12.5 min	Enhancement in solubility and foaming capacity and reduction in particle size	Increase in the interaction between protein and water molecule so larger surface of protein concealed	[157]
Myofibrillar proteins	100 and 300 W, 20 kHz	30 min	Improvement of emulsifying and rheological properties	Changes in α-helix, β-sheet, β-turn, and random coil con- tents	[158]
Dietary corn and rice proteins	800 W, 20 kHz	10 min	Effectively improve the bioavailability of dietary proteins and increase the content of 200–1000 Da peptides	Changing protein structure and composition	[79]
Stevia protein hydrolysate	300 W, 50 kHz	30 min	Significant influence on ACE inhibitory activity.	Expose more hydrogen bonds during ultra-sonication	[159]
Antidiabetic peptides	500 W, 20 kHz	15 min	Improved the release of bioactive peptides	Cleaving weak bonds	[160]

### 5.1 Plant-based proteins

Plant proteins are rich sources of essential amino acids, minerals, and vitamins, low in calories and fat content. Besides nutritional properties and high antioxidant activity plant, proteins are also cheap; therefore, their consumption is enhanced over time [80]. The bioavailability of many proteins, especially plant proteins, is low due to dense and tertiary structures, which waste many proteins [81]. Various methods are used to enhance the bioavailability and digestibility of proteins, including chemical and non-chemical methods, but so far, HIU is the best option as it can have both mechanical and chemical effects and is non-toxic and safe [9], [82].

In a study, ultrasonication was used to improve the bioavailability of daily consumed dietary proteins (rice, soy, corn, and oat). Results suggested that during in vitro digestion, there was a significant increase in HIU-treated proteins. All four treated proteins' digestibility was enhanced by 9.49% (rice), 9.97% (oat), 8.19% (corn), and 9.84% (soy), respectively. The caco-2 model also revealed that the absorption of HIU-treated proteins significantly increased compared to the control. There was also an increased content of 200–1000 Da peptides [83]. Similarly, in another research, buckwheat protein isolates were subjected to HIU (20 kHz with an amplitude of 60% and time duration of 10 min). The in vitro digestibility analysis showed a 16% increase in digestibility, and the effect of HIU on tertiary analysis showed the exposure of a hydrophobic core buried inside the molecule and a significant decrease of ß-turn and ß-sheet [84].

Due to their nutritional, medicinal, functional, and physiological properties, potato proteins are used in many food applications and have recently gained more importance in the food industries. Potato proteins are considered non-allergic and GRAS [85]. In a study, potato protein was treated with HIU 600 w for 20 min. A pH drop method was used to investigate the effect of HIU on bioavailability; the results revealed that ultrasound-treated potato protein had a 16 % increase in bioavailability. The increase in bioavailability resulted from exposure to hydrophobic residues (Hussain et al., 2021). In recent research, chickpea protein was subjected to different ultrasonic power (200, 400, 600 W) for different times (10, 15, and 30 min). HIU treatment showed alterations in surface hydrophobicity, indicating HIU treatment exposed more hydrophobic amino acids and released more negatively charged groups resulting in enhanced digestibility [86]. It is suggested that ultrasound has great potential and is conducive to enhancing the bioavailability and digestibility of animal-based and plant proteins [87].

### 5.2 Animal-based proteins

Animal-based proteins (meat and its derivatives) are a rich energy source for growth and maintenance [88]. Fresh meat generally has high digestibility and bioavailability, but the meat derivatives, and upon storage, meat loses its digestibility and quality [89], [90]. Different methods are applied to maintain animal-based protein's quality, bioavailability and digestibility, including high temperatures, acids, and electric pulse. Among these, ultrasonication is considered a wise option for treatment [91], [92].

A study [91] examined the effect of HIU (400 W, 20 kHz) on the digestion of pork meat extracted actomyosin during storage. The structural and chemical alterations were determined through spectroscopic methods. Results showed a continuous decrease in digestibility during storage time; HIU-treated samples were significantly more digestible for all treatments. The results obtained during in vitro digestibility were supported by those obtained from SDS and LC-MS, indicating high proteolysis during HIU treatment. The increase in digestibility was due to the unfolding and reduced interaction of amino acids [91]. Likewise, a study was conducted to check the effect of HIU on the bioavailability of animal protein (pork) myofibril. With the storage time, the bioavailability of myofibrillar decreases. Obtained results showed that HIU treatment significantly increased the bioavailability and also helped produce total peptides count by reducing β-turn and α-helix contents [93].

Numerous factors influence the effect of HIU on the bioavailability and digestibility of proteins, including freezing, cooking and other chemical treatments [94]. The effect of HIU on the chicken myofibril emulsion gel with polyphenol was studied. Chicken myofibrils were extracted from chicken breast, and polyphenols (epicatechin gallate and baicalein) were obtained from Chinese green tea; polyphenols and chicken myofibrils were attached through covalent bonding with adjustment in the pH. The mixture was treated with HIU (350 ± 20 W/L for 6 min). HIU treatment and the presence of Epicatechin gallate significantly enhanced myofibril's digestibility and reduced protein aggregation during digestion. HIU and Epicatechin gallate synergistically works to enhance digestibility by unfolding protein and increasing hydrophobicity and active sulfhydryl content. It also improved antioxidant activity [95].

Similarly, [96] investigated the impact of HIU (40 kHz) on the digestion and freezing of chicken breast. Samples were thawed, cooked, and subjected to in vitro digestion and compared with untreated samples; results indicated that ultrasonication effectively reduced the freezing time by 11% and significantly increased the degradation of proteins during the gastrointestinal phase. There was also a substantial increase in the number of peptides in HIU-treated samples compared to untreated.

For the formulation of infants, puree HIU has also been used to enhance the digestibility of offals. The effect of HIU on the bioavailability of pork liver was studied in research. Liver samples were treated with ultrasound (265 W, 42 min) and then subjected to in vitro digestion, and digestibility was calculated as percent nitrogen emission. The analysis showed untangling of proteins and increased hydrophobicity; these alterations significantly enhanced the digestibility of treated samples [97], [98].

Fish and seafood are rich sources of nutrition; several techniques, including HIU, are used to enhance the digestibility of these proteins [99]. The impact of HIU on the digestibility of shrimp protein was evaluated; samples were treated with (20 kHz for 400 W at different times) and then analyzed under gastrointestinal digestion. The results revealed changes in the secondary structure. Samples treated for 20 min were significantly more digestible [100].

Canned pāua, Haliotis iris, is protein-rich seafood. It is the finest product made in New Zealand research. It was conducted by [101] to examine the impact of HIU treatment on the digestibility of pāua. HIU was applied on the whole paua meat with the intensity of (20 kHz, 464 ± 9 W) for 5 min. Post-treatment cooking of canned pāua was done in a water retort at 116 °C for 30 min. The results showed lower slice shear force values and increased tenderness in HIU-treated samples compared to untreated. The enhancement of tenderness in the treated samples was due to the dissociation of myofibers and the formation of pores between myofibers, as shown in histological analysis and S.E.M.[101]. Ultrasonication significantly impacts the bioavailability and digestibility of protein extracted from marine and freshwater species, including shrimps, soft-shelled turtles, and New Zealand *Abalone* (*Haliotis iris*) [102], [103]. In conclusion, animal- and plant-based proteins can be treated with H.I.U. to enhance bioavailability and digestibility. Numerous pieces of research suggest that HIU treatment can significantly enhance the bioavailability of proteins; although there are some limitations, such as discoloration and flavor-related changes mentioned in the literature, it is negligible.

## 6 Role in protein allergenicity

One of the biggest problems with the consumption of proteins is an allergy, especially proteins found in some nuts (peanuts, hazelnut) and also in seafood (shrimp, crab, and shellfish) [104], [105]. Common symptoms associated with protein allergies are respiratory, gastrointestinal, cardiovascular, and anaphylaxis in some most severe cases [106]. HIU is recently considered to have the ability to reduce the allergenicity of different allergy-causing proteins. During HIU treatment, high-energy waves cause physical and chemical changes in formation and collapsing bubbles, producing conformational changes in allergens and reducing their reactivity [107], [108], [109]. The cavitational-induced alterations by ultrasonication in allergenic proteins affect antibodies' capability to react with modified proteins, thus decreasing the incidence of IgE-induced food allergy reactions [110]. Also, high shear force disturbs hydrogen bonding and van der Waals forces, causing protein denaturation and low allergenicity.

To reduce the allergenicity in different foods, varying frequencies and treatment time is required. For example, different frequency is required to treat soy flour, peanut, and milk. In shrimp, ultrasonication can reduce allergenicity from 100% to 25%, showing the technique's potency against allergens [100]. Different studies have proven that dissociating high molecular proteins to lower peptides reduces allergenicity and enhances bioavailability [111].

The prevalence of Allergy incidence by milk protein is approximately 5–10 % of the population of infants and children and has become a significant public safety problem [112], [113]. In cow milk, the main allergy-causing proteins found are casein (CN) and lactoglobulin (LG) [114], [115]. Research conducted in 2022 by [116] investigated the effect of ultrasound on lactoglobulin (L.G.) and luteolin (LT) and examined the effect on allergenicity and human intestinal microbiota. The ultrasonic treatment produced conformational changes, decreased hydrophobicity, and increased non-covalent interactions by hydrogen bonding. Furthermore, ultrasound treatment has reduced IgG/IgE binding capacity and prevents allergic reactions of KU812 cells depending on conformational changes.

Similarly, in another investigation, fresh cow milk and casein isolated from cow milk were treated with high-intensity ultrasound (25 kHz frequency, 900 W) for 30, 45, and 60 min. It was found that HIU treatment drastically decreased CN's particle size to <100 nm. The TEM and electrophoresis also showed significant changes in the protein structure. Furthermore, serum-containing enzyme-linked immunoassay exhibited a substantial decrease in the immunoglobulin-E-binding capacity of CN. Similarly, the LAD2 mast-cell line degranulation assay showed hypo-allergenicity of samples treated with HIU in CN and fresh milk [117].

Pru p3 is an important cause of plant food allergy, an allergen found in plant-based products, especially peaches. It can cause severe allergic reactions in certain people [118]. The impact of ultrasonication and heat treatment on allergenicity and denaturation of Pru p3 has been studied; the allergenicity was estimated by an immune fluorescent assay using peach allergic individuals sera, and the amount of denaturation was investigated through sandwich ELISA. The results showed a 60 percent denaturation of Pru p3 protein, which was treated at 95 °C for 40 min. Conversely, ultrasound and heat treatment at the optimum conditions have reduced the IgE-binding ability below 10 % [119].

Likewise, in some people, kiwifruit can cause an allergy that can even lead to death [120]. In a study, HIU was used to treat kiwifruit samples (20 kHz, 400 W, 50%) for 16 min to check its effect on the allergenicity of kiwi fruit allergen Act d2. The results revealed that HIU produced resounding alterations in kiwifruit tissues, resulting from changes in the secondary structure, including the beta-sheet and alpha helix. HIU treatment for 16 min caused a 50 % reduction in kiwifruit allergen Act d2 and improved in vitro digestibility by up to 62 %. Similarly, HIU treatment also decreased the solubility of kiwifruit total proteins by 20 % [121].

## 7 Role in anti-nutritional factors

Cereals and legumes are rich sources of nutrients, especially proteins, and provide a fair amount of energy but also contain anti-nutritional factors [122]. Major anti-nutritional factors in consumable Food are lectins, saponins, gossypol, tannins, and protease inhibitor [123].

Anti-nutritional factors in Food attach to nutrients and reduce their bioavailability. Some anti-nutritional factors in legumes and cereals suppress protein digestibility and mineral absorption [124], [125]. Anti-nutrients can reduce the absorption of various nutrients, which can cause micronutrient malnutrition and mineral deficiencies [126].

Several techniques can be applied to reduce anti-nutritional factors, including soaking, germination, autoclaving and fermentation. The application of ultrasonication in reducing anti-nutritional factors has recently been reported [127], [128]. Tannin can precipitate food proteins and change them into anti-nutritional factors, reducing Food's bioavailability. HIU treatment with increasing amplitudes and time can reduce amounts of tannin in Food [128], [129].

Elephant foot yam is a rich source of nutrients, including protein, it also contains plenty of micronutrients like potassium, calcium, and vitamin C, but due to the presence of anti-nutritional factors like oxalates and acridity, it's under-consumed. Ultrasonication can alter the protein structure; therefore, it might be a promising technique for the reduction of anti-nutritional factors efficiently. The temperature generated during ultrasonication denatures anti-nutritional factors resulting in reduced activity [130].

Finger millet is a rich source of protein and minerals, the amount of calcium and iron in finger millet is greater than other cereals, but it also contains anti-nutritional compounds like phytates and tannins [131], [132]. Conventionally, the hydration process removes these anti-nutritional factors with poor product outcomes. Research conducted by [133] applied HIU with an amplitude of 66% to finger millet grains for 20 min. The results showed a significant reduction in phytates 66% and tannins 62.83%. HIU treatment increased water binding capacity and solubility in millet grains, and XRD also showed enhancement in the crystallinity of starch particles and size reduction [133].

## 8 Enhancement in functional properties

**Fig. 3:**
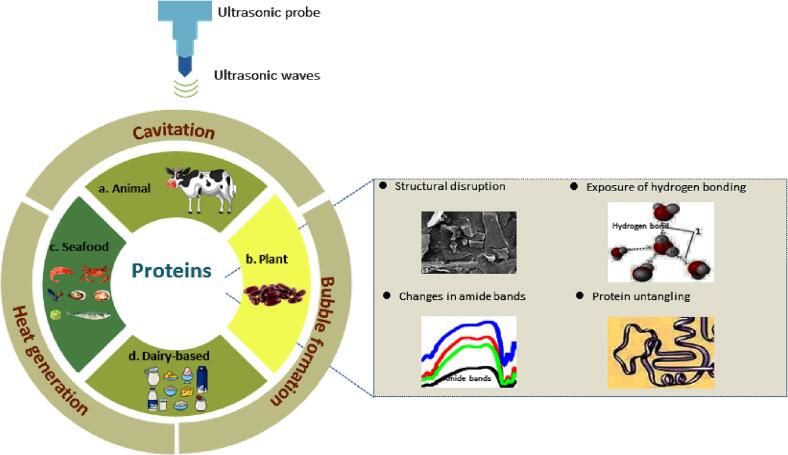
Presenting the impact of HIU structural aspects of different proteins.

A protein's functional properties define its quality; any protein containing better functional attributes is considered high-quality [134]. The functional properties of proteins and protein-rich Food are defined by their structure, which ultrasonication can modify. Therefore, ultrasonication can easily alter the functional properties of proteins by changing the structure [135]. The impact of HIU treatment on the functionality of different proteins is presented in Fig. 3. Different kinds of HIU-treated proteins enhanced functional properties, including foaming ability, hydrophobicity, and emulsifying activity; hence, HIU-treated proteins can be efficiently used as functional ingredients in food and nutraceutical industries [136].

In a research plant, proteins isolated from agriculture by-products (black soybean) were treated with HIU to enhance functional properties. The extracted protein was treated with 40 kHz at 350 W for (20, 40, and 60 min). It was noticed that ultrasonication treatment altered secondary structure decreased α-helix and β-sheet contents, and exposure of polar and nonpolar amino acid residues. Similarly, HIU enhances free sulfhydryl amount and decreases particle size, increasing foaming ability and capacity, solubility, and emulsion stability [137].

Due to the explosive population growth, the need for healthy and nutritious protein is increasing; therefore, recently, plant-based proteins are gaining importance [138]. But most plant-based proteins have poor solubility and lower foaming and gelling properties [139]. In a research, hemp seed protein (a great source of unique amino acids) was treated with ultrasonication (600 W 5 min) and pH adjustments. The results showed that treated samples with ultrasound increased solubility by 19 % and similarly enhanced the volume of free sulfhydryl content (*p* < 0.05) to 32.8 mmol/g [140].

Seed proteins are considered highly nutritious and bioactive and used for different purposes, including extraction of bioactive peptides [141]. In a study, proteins extracted from apple seeds were subjected to ultrasonication to analyze the impact on the functional properties. Ultrasonicated samples were characterized for size, structure, and functional properties, SEM revealed obvious alterations in the surface morphology, and DSC showed a decrease in denaturation temperature. All treated samples significantly improved functional properties, including solubility, hydrophobicity, foaming ability and emulsifying properties [136]. Likewise recently, insect proteins have been considered one of the finest proteins with the highest nutritional value. Tussah pupa protein is considered an alternative to traditional plant and animal-derived proteins [142], [143], but the processing potential is meager. Research conducted by [144] to evaluate the effect of HIU on the functional properties of pupa protein isolates. Samples were treated for 100 min with an amplitude of 40 %; the results showed that compared with untreated samples in HIU treated samples, there was a significant 4.57 times increase in solubility, emulsifying stability, and emulsifying activity 0.23 and 2.10 times, respectively, and 1.83 times in foaming stability. In addition, elasticity and surface hydrophobicity also significantly improved. Protein structure analysis showed a reduced di-sulfide bond and increased beat sheet content [144].

The research mentioned above supports the application of ultrasonication as a promising alternative to the traditional and chemical methods used to modify the functional properties of protein and widen their application in the Food, cosmetic, and nutraceutical industry.

## 9 Limitations

Besides all the favorable research data and application of ultrasonication in food research and food industries, it has still various limitations to consider. Following are some of the limitations mentioned.

a) Choosing ultrasound frequency and time is critical; different proteins have different structures and functionality; therefore, a specific frequency and time can't be applied for every protein.
b) The generation of enormous heat, especially at a continuous scale, is a significant disadvantage.
c) When applied on industrial levels, the pilot scale results sometimes do not generate similar yields.
d) There are few, but some research mentioned undesirable protein flavor development after ultrasonication.

## 10 Future prospects

As a green and physical technique, high-intensity ultrasound has several benefits in altering protein structure to enhance bioactivity, bioavailability, and other functional attributes, which have been investigated and documented by numerous investigators. However, many shortcomings still need to be addressed for the enhanced application of HIU to modify different protein attributes.

(1) Recent techniques, including molecular dynamics simulations and proteomics, should be acquainted with to unfold the actual mechanism of HIU Induced structural changes in protein and its impacts on nutrition bioavailability and digestibility.
(2) For the bioavailability of proteins after HIU treatment, an animal model or in-vitro digestion is not ideal; therefore, developing a technique to analyze the bioavailability and bioaccessibility of proteins inside the human body is necessary.
(3) Most of the research regarding the impact of HIU on bioactivity and bioavailability of protein has been done on a small scale. Therefore, developing an upscale model of HIU apparatus that can be used at the industrial level is further required.
(4) During ultrasonication, an enormous amount of noise is generated by the waves, which needs to be reduced for the safety and health of workers.
(5) There is sufficient data on the impact of HIU on the modification of plant-based and animal-based proteins but little knowledge on proteins derived from insects, aquatic animals, and proteins derived from agricultural wastes, which need to be analyzed in the future.

## 11 Conclusions

Ultrasound is acclaimed by researchers for its desirable impacts on bioactivity, bioavailability, functional properties, and overall quality of edible proteins. Also, it doesn't need chemical treatment to assist the technique, as HIU has both chemical and mechanical effects on protein. Concurrently, HIU treatments can alter digestible, structural, and nutritional attributes. Several investigations suggested that HIU treatment can substantially increase bioactive peptides' antioxidant activity, antimicrobial activity, and anti-cancer capacities. Besides, HIU can help reduce proteins' allergenicity and anti-nutritional factors without altering nutritional properties. In a nutshell, ultrasonication is a wise option to enhance protein quality. Yet HIU is not universal that improves all aspects of protein; for example, not a specific time and amplitude can improve the quality of different proteins. Also, large-scale studies are still limited; more investigations are required for the practical implication of HIU at the commercial level.

## CRediT authorship contribution statement

**Muhammad Hussain:** Conceptualization, Methodology, Writing – original draft, Writing – review & editing. **Munkh-Amgalan Gantumur:** Writing – original draft. **Muhammad Faisal Manzoor:** Data curation, Writing – original draft, Writing – review & editing. **Kifayat Hussain:** Data curation, Writing – original draft. **Jie Xu:** Data curation, Writing – original draft. **Rana Muhammad Aadil:** Data curation, Writing – original draft. **Abdul Qayum:** Data curation, Writing – original draft. **Ishtiaq Ahmad:** Data curation, Writing – original draft. **Hao Zhong:** Supervision, Writing – review & editing, Funding acquisition. **Rongfa Guan:** Supervision, Writing – review & editing, Funding acquisition.

## Declaration of Competing Interest

The authors declare that they have no known competing financial interests or personal relationships that could have appeared to influence the work reported in this paper.

## Data Availability

The authors do not have permission to share data.
